# Importance of genomic surveillance of SARS-CoV-2 in cats during reverse zoonosis events: potential viral evolution may occur

**DOI:** 10.1128/spectrum.00680-23

**Published:** 2023-08-11

**Authors:** Sandra Barroso-Arévalo, Marta Díaz-Frutos, Lucas Domínguez, José M. Sánchez-Vizcaíno

**Affiliations:** 1 VISAVET Health Surveillance Center, Complutense University of Madrid, Madrid, Spain; 2 Department of Animal Health, Faculty of Veterinary, Complutense University of Madrid, Madrid, Spain; Oklahoma State University College of Veterinary Medicine, Stillwater, Oklahoma, USA

**Keywords:** SARS-CoV-2, cats, mutation, genomic surveillance, viral evolution

## Abstract

The apparition of new variants of severe acute respiratory syndrome coronavirus 2 and lineages is constantly happening because of the high viral mutation rate. Since numerous reverse zoonosis events have been reported so far, genomic surveillance should be conducted in susceptible species to evaluate potential adaptations that may trigger the apparition of new variants. Here, we evaluate the evolution of the infection in a cat naturally infected in parallel with its owner, performing a comparative phylogenetic analysis. Sequencing analysis showed that both were infected with the Omicron BA.5/BF.1 lineage and revealed the presence of nucleotide substitution in the viral genome recovered from the cat with respect to the viral genome from the human sample. This nucleotide substitution (C11897A) produced the amino acid change Orf1a: Q3878K. Therefore, genomic surveillance in the case of reverse zoonosis events is still necessary in order to control possible adaptations of the virus to other susceptible species.

**IMPORTANCE:**

Genomic surveillance of pets for severe acute respiratory syndrome coronavirus 2 is important to monitor the emergence of new variants of the virus associated with these animals. Pets can serve as a potential reservoir for the virus, and their close contact with humans increases the risk of transmission. By conducting genomic surveillance in pets, it is possible to detect and track new variants early on, allowing for more effective control measures to be put in place. This can help prevent the spread of these variants to human populations and potentially mitigate the impact of the pandemic. Furthermore, it may also provide insight into the evolution and spread of the virus within the animal population.

## INTRODUCTION

After more than two years, the pandemic triggered by the severe acute respiratory syndrome coronavirus 2 (SARS-CoV-2) is still active. Although the disease control measures have been relaxed in the last months, the virus is still active, as reflected by the high levels of infections (1). Luckily, the fatality of the cases has decreased substantially due to the increase in vaccine coverage and infection-acquired immunity in the human population by other variants (2, 3). However, it is crucial to account for additional factors, such as the emergence of new variants, variations in public health measures, and differences in vaccine efficacy against various strains. Several variants of concern (VOCs) have emerged during these years of the pandemic. On November 26, 2021, the World Health Organization declared the latest VOC of SARS-CoV-2, the Omicron (B.1.1.529) variant initially detected in South Africa. In the following months, Omicron spread continuously over the world, replacing the previous dominant variant, Delta (B.1.617.2). Subsequent experiments proved that Omicron presented a higher infectivity rate and a great capacity to evade current vaccines and previous immunity (4). This suggests that SARS-CoV-2 is still adapting to its human host.

Five lineages have been described in the case of Omicron: BA.1 (B.1.1.529.1), BA.2 (B.1.1.529.2), BA.3 (B.1.1.529.3), BA.4 (B.1.1.529.4), and BA.5 (B.1.1.529.5). In Spain, the Omicron BA.5 (B.1.1.529.5) lineage is currently the dominant variant. This isolate presents an even higher transmission capacity but with less severe disease outcomes than the previous ones (5) and is then responsible for the increase in cases in the country since May 2022 (1). BA.5 carries its unique mutations, including changes called L452R and F486V in the viral spike protein that might tweak its ability to latch onto host cells and skirt some immune responses. Several sublineages of BA.5 have emerged, such as BF.1, first detected in Asturias, Spain (6). This sublineage has been gaining rise in the United Kingdom, Scotland, Denmark, and Canada, as well as in the North of Spain (7). Little is known, however, about the susceptibility of this new subvariant of Omicron in other nonhuman susceptible species such as cats. According to experimental and field studies conducted on pets, SARS-CoV-2 seems to produce anecdotic infections in them, although several VOCs have been related to more severe clinical cases or higher viral loads (8 – 11). Although previous works performed by our research group showed that susceptibility to Omicron BA.1 lineage is quite low in pets (12), potential adaptation to new hosts cannot be ruled out in the case of other lineages. Here, we describe the evolution of the infection of a cat with the Omicron subvariant BF.1 in parallel with the evolution of the infection in the human host, who acted as a source of infection. The cat became infected two days after exposition to the infected human and maintained positive results for RT-qPCR for four days. The only clinical signs presented by the cat were occasional sneezes. Importantly, sequencing results showed a nucleotide substitution in the sequence from the cat with respect to the human’s sequence. This nucleotide change was located in the ORF1a protein-coding region. This work has demonstrated for the first time that potential genomic adaptations can occur in the case of reverse zoonosis events in cats, which highlights the importance of performing active surveillance on domestic animals exposed to infected people.

## MATERIALS AND METHODS

### Animal and human sampling and clinical inspection

Human SARS-CoV-2 infection was confirmed by a SARS-CoV-2 antigen test (Roche, Basel, Switzerland) and RT-qPCR on 24 May 2022 after showing COVID-19-compatible symptoms (fever, fatigue, and headache). Cat and human samplings were daily conducted from this day until 10 days after that. Samples consisted of oropharyngeal (cat) and nasopharyngeal (human) swabs collected in DeltaSwab Virus 3 mL contained in viral transport media (VTM) (Deltalab S.L., Cataluña, Spain) using protocols approved by the Complutense University of Madrid’s Ethics Committee for Animal Experiments (Project License 14/2020). Serum sample from the cat was collected in a tube without any anticoagulant 14 days after the initial cat sampling in order to evaluate the presence of neutralizing antibodies. During the sampling period, the cat was daily observed in search of clinical signs, and pulmonary auscultation was performed by a veterinarian.

### Detection of SARS-CoV-2 infection by reverse transcription-quantitative PCR

Total RNA extraction and reverse transcription-quantitative PCR (RT-qPCR) were performed as previously described in reference (13).

### Virus and cells

SARS-CoV-2 MAD6 isolated from a 69-year-old male patient in Madrid (Spain) was kindly provided by Dr. Luis Enjuanes from the National Biotechnology Centre at the Higher Council for Scientific Research.

Vero E6 cells (ATCC, Manassas, VI, USA) were prepared in order to reproduce the SARS-CoV-2 stocks. Cells were incubated at 37°C under 5% CO2 in Gibco Roswell Park Memorial Institute (RPMI) 1640 medium with lglutamine (Lonza Group Ltd., Basel, Switzerland) and supplemented with 100  IU/mL penicillin, 100  µg/mL streptomycin, and 10% fetal bovine serum (FBS) (Merck KGaA, Darmstadt, Germany). SARS-CoV-2 titers were determined via a tissue culture infectious dose (TCID50) assay.

### Neutralizing antibody detection

Serum was tested for neutralizing antibodies against SARS-CoV-2 using the SARS-CoV-2 surrogate virus neutralization test (GenScript, Inc., NJ, USA), according to the manufacturer’s instructions. This kit has been previously validated in references 13 – 15.

### Viral isolation

Positive specimens from the cat and the human were subjected to virus isolation. Viral isolation was performed in six-well plates with monolayers of Vero E6 cells at 80% confluence. The cell medium was removed, and the inoculum containing 200 µL of the sample plus 800 µL of free FBS medium was added. Then, adsorption of the virus was performed in continuous agitation at 37°C for 1 h. After that, fresh medium with 5% FBS was added. The plates were incubated at 37°C with 5% CO_2_ for 5 days. The plates were daily observed, finding a cytopathic effect. After 5 days, the cell cultures were frozen, thawed, and subjected to three passages with inoculations of fresh Vero E6 cell cultures with the lysates, as described above. SARS-CoV-2 molecular detection was performed by employing RT-qPCR on the supernatants from every passage in order to confirm the presence/absence of the virus in the cell culture and virus recovery by means of the decrease in the Ct.

### Whole-genome sequencing and phylogenetic analysis

Whole-genome sequence was obtained from the positive oropharyngeal (cat) and nasopharyngeal (human) samples (both biological samples) with the lowest Ct value by RT-PCR using the methods previously described in reference (13) using Sanger sequencing, a method particularly efficient in correctly reading through homopolymeric regions (16).

MEGA 11 software (17) was used for the phylogenetic analysis. The analysis involved both sequences obtained in this study (cat and owner) as well as representative sequences from Spain, including the five lineages of the Omicron variant (BA.1, BA.2, BA.3, BA.4, BA.5, and BF.1). The final alignment involved 22 whole-genome sequences, with an average amino acid p-distance (1-amino acid identity) of 0.011, which is considered adequate since it is within the acceptance threshold of <0.8. This alignment was used to build the phylogenetic tree using the maximum likelihood method, the Subtree-Pruning-Regrafting (SPR) algorithm, and bootstrap testing of 2,000 replicates. Since only those bootstrap values ≥70% are considered valid, a consensus tree was computed, accepting the default 50% cut-off value, according to Hall, BG (18), in such a way that several clades are shown as a polytomy.

The presence of mutations was evaluated using the CoVsurver mutations app and AudacityInstant (v3.08) available on the GISAID website (https://www.gisaid.org/) (accessed on 30 September 2022). We appreciate and acknowledge the different laboratories and funders of GISAID for offering these SARS-CoV-2 sequences. A comparative analysis of both sequences was also performed using Nextclade (https://clades.nextstrain.org).

### Prediction of mutation significance in the protein

The sequence of the nsp7 SARS-CoV-2 protein was retrieved from GenBank-protein (NCBI Reference Sequence: YP_009742614.1) as a reference. The sequences corresponding to the same protein from both the owner and the cat (mutated protein) were obtained using Expasy (https://web.expasy.org/translate/). The MudPred2 tool (http://mutpred.mutdb.org/) was employed to analyze the amino acid sequences of two proteins: the wild type (from the owner sequence) and the mutant variant (from the cat). MudPred is a computational tool that predicts the impact of amino acid substitutions on protein stability and function. The analysis was performed by inputting the amino acid sequences of the proteins and running the MudPred algorithm. The output of the analysis included the site of the mutation, the MudPred score, and the predicted mechanisms that could be impacted by the mutation (19). Values of *P* < 0.005 were considered significant.

## RESULTS

### Clinical case description

The cat, a common European 2-year-old cat, had been living with a confirmed COVID-19-positive owner since 24 May 24, 2022. The owner reported a high rate of close contact (kisses, sleeping together, and coughing in the presence of the animal). The only clinical sign displayed by the cat was occasional sneezing.

### Comparative evolution of the infection between the cat and the owner

**Fig 1:**
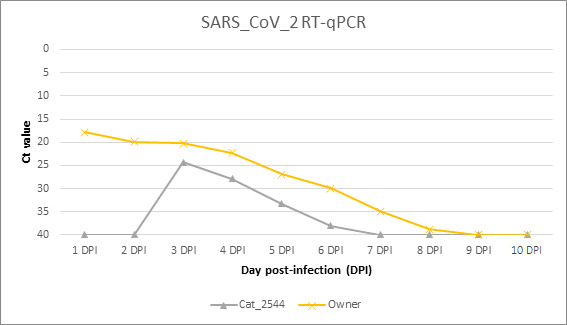
Comparative Ct (cycle threshold) values for SARS-CoV-2 RT-qPCR in the cat and its owner.

The cat became infected and showed the first positive result for RT-qPCR 2 days after the owner’s disease confirmation. While the human resulted positive for RT-qPCR for 8 days, the cat was positive for 4 days, with lower viral loads than the owner. Infection evolution is shown in Fig. 1.

Virus isolation was possible from the owner’s first sample (Ct value: 17.89). However, virus isolation was not possible from any of the cat’s samples.

### Neutralizing antibodies detection by employing VNT

Serum sample taken from the cat 14 days after the initial sampling presented neutralizing antibodies according to the SARS-CoV-2 surrogate virus neutralization test, with an inhibition percentage of 86.

### Whole-genome sequencing and phylogenetic analysis

Two sequences were obtained in this study, one from the cat (EPI_ISL_16456631) and the other from its owner (EPI_ISL_15809378). Both sequences were identified as VOC Omicron GRA (B.1.1.529 + BA. 2.1.1) BF.1 lineage, according to AudacityInstant (GISAID). Regarding the owner’s sequence, there were 144 related genomes found (at a distance of 2 or less from the uploaded sequence). The minimum quality of the matches was 0.901. Among the related genomes, the most frequent country was the United States (32.6% of genomes), the most frequent lineage was BF.1 (57.6% of genomes), and 81.1% of the related genomes were from samples collected between June 2022 and August 2022. In the case of the cat’s sequence, there were 177 related genomes found (at a distance of 2 or less from the uploaded sequence). The minimum quality of the matches was 0.909. Among the related genomes, the most frequent country was the United Kingdom (47.5% of genomes), the most frequent lineage was BF.1 (44.1% of genomes), and 81.8% of the related genomes were from samples collected between May 2022 and August 2022.

**Fig 2:**
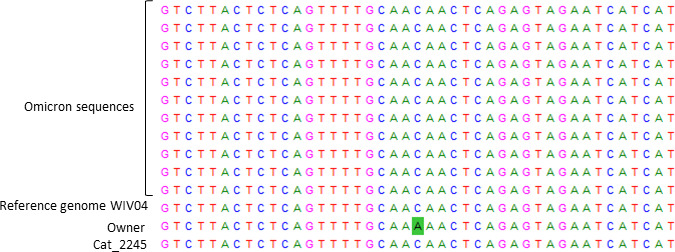
Alignment of the partial nucleotide sequence of the ORF1ab protein of SARS-CoV-2 showing the nucleotide change (C is replaced by an A) in the position sequence of Cat_2245 with respect to the owner´s sequence. Ten additional Omicron sequences (EPI ISL 13824643, EPI ISL 13833466, EPI ISL 13844673, EPI ISL 13876324, EPI ISL 13907952, EPI ISL 13907956, EPI ISL 13967718, EPI ISL 13967733, EPI ISL 13970526, EPI ISL 13986409) were included in the analysis as well as the reference SARS-CoV-2 sequence (WIV04).

Comparative analysis of both sequences revealed one change in the nucleotide sequence of the cat compared to the owner’s sequence. The change occurred in the nucleotide position 11,897 (located in the ORF1ab), where a C was replaced by an A (Fig. 2). This nucleotide substitution involves the amino acid substitution Orf1a: Q3878K.

**Fig 3:**
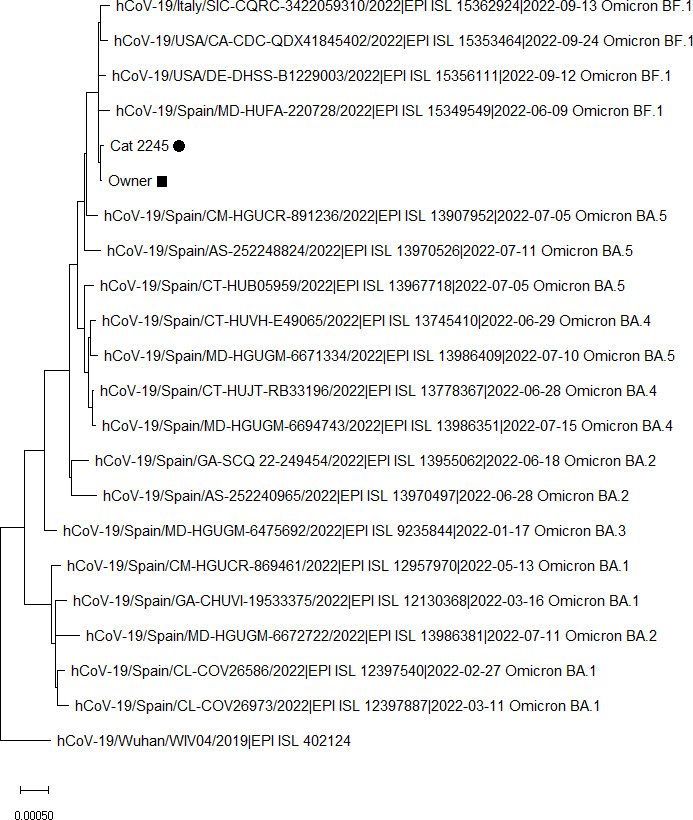
Phylogenetic analysis of SARS-CoV-2 (severe acute respiratory syndrome coronavirus 2) indicated that the whole-genome sequences from this study (black square: Owner; black circle: Cat_2245) clustered with the genomes of the SARS-CoV-2 BF.1 (Omicron) sublineage that were included in the alignment. Reference SARS-CoV-2 (WIV04) genome from Wuhan was also included. We appreciatively acknowledge the different laboratories and funders of GISAID for offering these SARS-CoV-2 sequences.

The maximum likelihood based on the Tamura–Nei model (20) was used for inferring the evolutionary relationships among the different whole-genome sequences. A total of 22 nucleotide sequences, including first, second, third, and noncoding codon positions, were analyzed. In order to avoid the inclusion of alignment gaps, missing data, and ambiguous bases, positions with <95% site coverage were removed from the alignment, resulting in the analysis of 29,728 positions. The evolutionary history of the analyzed sequences was obtained from the bootstrap consensus tree deducted from 2,000 replicates (17). First, initial tree(s) were obtained automatically using the neighbor-joining and BioNJ algorithms to a matrix of pairwise distances estimated using maximum composite likelihood and then choosing the topology with a better log-likelihood value. For the modelization of differences in the rate of evolution among different sites, a discrete gamma distribution (two categories, +G parameter = 0.067) was used. The resulting phylogenetic tree is shown in Fig. 3. As observed in the phylogenetic tree, the genome sequences from this study (Cat_2245 and Owner) clustered with sequences belonging to the BF.1 (Omicron) sublineage.

### Mutation significance

The results of the MudPred analysis indicated that the mutated protein (from the cat) carries a substitution at site Q19. The MudPred overall score for the mutated protein was 0.30894, indicating a relatively low probability of the mutation being pathogenic. The predicted mechanisms impacted by the mutation include the catalytic site (*P* = 0.028316), iron binding (*P* = 0.039549), and proteolytic cleavage (*P* = 0.040032).

## DISCUSSION

Numerous reports have evidenced the capacity of SARS-CoV-2 virus to produce reverse zoonosis events (13, 21 – 23). Despite the increased adaptation to human receptors that is expected to have occurred during virus evolution, susceptible animal species remain infected when exposed to infected humans or their excretions. This fact evidences the necessity to perform surveillance in these species in order to detect potential virus alterations derived from different hosts. Here, we have studied the SARS-CoV-2 infection evolution in an infected cat in parallel with its owner, who acted as the source of infection for the animal. In addition to RT-qPCR monitoring of both human and pet, sequencing analysis was performed, showing potential adaptation of the virus to the animal host, as reflected by the presence of a nucleotide substitution.

Both the owner and the cat in this study were infected with the Omicron BF.1 sublineage of the BA.5 variant, which has been quietly replacing the previous Omicron strains (24). According to current knowledge, BA.5 and its derivated lineages are the predominant lineages in Spain (25), representing 84.7% of the sequenced samples in the country. Regarding genome sequencing and evolutionary analyses, BA.5 and their sublineages appear to have developed enhanced pathogenicity, transmission ability, and the potential of escaping neutralizing antibodies induced by vaccination or infection (26). Concretely, BF.1 comprises some exclusive mutations in its S protein, including nsp16_V288F and nsp6_V289L, which confer greater fusogenicity and resistance to the immunity triggered by previous infection with early variants or vaccination. Additionally, a recent study has reported that BA.5 is still low pathogenic compared to the ancestral strain but has progressed to induce enhanced inflammation when compared to prior Omicron subvariants (26). All these together may explain the outcomes of this study, in which we have observed differences in the pathogenicity level of the BA.5 subvariant with respect to previous reports studying animal infection with the early BA.1 Omicron subvariant (12, 27). In our study, the cat was effectively infected after being exposed to the owner, and relatively high viral loads were detected in its oropharyngeal swabs during the first and second days of the infection, according to RT-qPCR results. This fact contrasts with a previous study conducted by our research group, in which active surveillance sampling was performed (12). In this previous report, carried out during the Omicron BA.1 wave in Spain, we evidenced a low susceptibility to the infection in the studied pets despite being in high contact with their positive owners. In fact, viral loads detected in the few positive animals were very low (Ct values above 30 in all the cases), and the shedding period was limited to one or two days at most. The outcomes of this previous study were also in line with those observed in an experimental study in Syrian golden hamsters (27), where lower replication levels of the virus were detected as well as a lack of typical histological lesions in hamsters infected with the BA.1 Omicron subvariant. On the contrary, the results presented here suggest higher pathogenicity of the BA.5 strain, similar to that observed with previous VOCs such as Alpha or Delta variants in pets (8, 9, 11), although further experimental studies should be conducted in order to confirm this hypothesis.

The most important conclusion from the present study is, however, the sequencing results that may indicate a potential for changes in SARS-CoV-2 when infecting cats. This fact has been previously suggested in the case of mink and deer (28, 29), but it has also been confirmed in the case of our study. This underscores the importance of performing genomic surveillance in all the susceptible species to SARS-CoV-2 infection. Our findings suggest that reverse zoonosis events might be associated with changes in the viral genome that may favor the apparition of new variants or increase the risk of transmission to other animals or even humans. While the efficacy of vaccines appears to be high in controlling the spread of the virus but still remains under debate, genomic surveillance of animal populations should be considered an important complementary measure. This would allow for early detection of potential viral adaptations and the implementation of targeted prevention measures, such as vaccination campaigns or targeted testing and isolation of infected animals. Further research is needed to identify the most effective strategies to control the emergence and spread of new viral variants, and we encourage the scientific community to continue to investigate this important topic. The rapid generation of nucleotide substitution in the virus infecting the cat contrasts with previous research that estimates the substitution rate of SARS-CoV-2 at around 1.16 × 10^3^ substitutions per site per year in the human population (30), which means around one mutation per 2 wk. RNA virus genomes are extremely predisposed to mutation, although the evolutionary mechanism by which the virus is capable of adapting to further mammalian hosts depends on the genetic variation generated within and between hosts. In a study conducted by Braun and collaborators (31), they demonstrated that species- and context-specific adaptations of SARS-CoV-2 are expected to continue to emerge. After inoculating three cats with SARS-CoV-2 virus and putting additional cats in touch to assess potential transmission, the authors evaluated genetic variations in the virus using whole-genome sequencing. With this approach, they observed limited within-host diversity. However, they found variants (S H655Y and E S67S) that were amplified in all three index cats on the first day post-inoculation. This predisposition of cats to variant selection has also been demonstrated in another study performed by Bashor and collaborators (32), where the authors detected up to 14 emergent variants in viruses recovered from animals during experimental infection. In addition, Klaus and collaborators (33) also observed single nucleotide polymorphisms in the viral genome sequence from the cat of study. These outcomes are in line with those presented here since the virus recovered from the cat in this study showed detectable genomic variation after infection, reflecting the capacity of the virus to generate mutations during the replication cycle in the host. The nucleotide substitution observed in this study was located in Orf1a, a gene that codes for nonstructural proteins (nsp1–16), responsible for the replication machinery and maintenance of the viral genome (34). Within this gene, the substitution was located in the region that codifies for the nsp7. This protein plays a key role in viral replication, working in tandem with the RdRp protein (RNA-dependent RNA polymerase) and the nsp8 protein, forming an RdRp–nsp7–nsp8 supercomplex (35). For this reason, mutations located in the nsp7 or nsp8 proteins may affect the complex formation among these proteins, consequently affecting genome replication (36). According to the analysis conducted to evaluate the significance of this amino acid change in the protein, it is presumed that the mutation may have had a low impact on the protein. Although our findings suggest the mutation might affect the protein’s function, these results must be interpreted with caution. Our study involved one SARS-CoV-2-infected cat, and while these findings contribute to our understanding of possible viral adaptations in this species, they are not conclusive due to the limited sample size. Thus, making any global claims regarding the evolution of SARS-CoV-2 in infected cats and the potential adaptation of this virus are not sustained by these data. However, empirical *P*-scores for each mechanism were considered significant. In this sense, disruption of the catalytic site, which is the specific region of an enzyme where the chemical reaction catalyzed by the enzyme takes place (37), was identified as the most probable mechanism for the mutation. It is, therefore, essential for the enzyme’s function, and its alteration can result in a loss of the ability to catalyze a specific chemical reaction by the involved enzyme. Another potential impact was the disruption of binding to iron. Iron is essential for many biological processes, such as DNA synthesis and the generation of ATP, and is crucial for viral replication within living host cells (38). In some viruses, such as the hepatitis C virus, iron plays a key role in viral replication cycle. By preventing the RNA polymerase NS5B enzyme from performing its normal enzymatic function, iron can inhibit the expression of viral RNA and proteins (39). Finally, mutations in the nsp7 protein could potentially affect its susceptibility to proteolytic cleavage. Proteolytic cleavage of viral proteins is also essential for viral replication and pathogenesis. All these mechanisms, therefore, are critical for the proper function of the protein, suggesting that the mutation may have a significant effect on the protein’s overall activity. Future studies will be needed to fully understand the impact of this mutation on the virus, and further *in silico* analysis may be conducted in order to explore the effect of this mutation on the interaction between nsp7 and RdRp proteins. It should be considered that mutations that give rise to substantial modifications in viruses typically tend to affect multiple nucleotides (40). Therefore, in this study, where only one nucleotide has been changed, the expected impact seems to be low. It could be hypothesized that the mutation found in this study may have been related to a decrease in the replication capacity of the virus since the cat in this study was able to remove the virus quickly (only 4 days). However, this hypothesis should be corroborated by more clinical case studies. Unfortunately, in studies reporting cases of reverse zoonosis, it is rare to have parallel sequencing of both the infected animal and the human acting as the source of infection. This has resulted in the inability to explore the occurrence of mutations associated with these events, and therefore, the results presented in this article must be approached with caution due to the low *n*. While our study focuses on a single case, its relevance lies in its potential to contribute to the broader understanding of SARS-CoV-2 host adaptation. We acknowledge that the extrapolation of our findings is limited. However, our study provides a crucial starting point for further investigations into SARS-CoV-2 infections and potential mutations in domestic cat populations.

In conclusion, the results presented here demonstrate that cats are also susceptible to the BA.5 Omicron and their sublineages. This lineage seems to be slightly more virulent for cats than the previous BA.1 strain, but still does not seem to represent a high risk for this species. Although the detected mutation in the SARS-CoV-2 nsp7 protein observed in the cat may suggest potential host adaptation, we want to clarify that this inference is made with caution due to the limited sample size of our study. The occurrence of the same mutation in additional individuals of the same species would provide stronger evidence of host adaptation. Our study serves to raise important questions about the potential for SARS-CoV-2 adaptation in various host species, a topic that warrants further investigation. We hope our work encourages further studies that include larger sample sizes and a broader range of host species to comprehensively address these questions.

## Data Availability

Whole-genome sequences from this study are deposited on the GISAID website.

## References

[1] Ministerio de Sanidad de España . 2022. Enfermedad por SARS-Cov-2 (COVID-19). Available from: ttps://www.sanidad.gob.es/areas/alertasEmergenciasSanitarias/alertasActuales/nCov/home.htm. Retrieved 16 Dec 2022.

[2] Dagan N , Barda N , Kepten E , Miron O , Perchik S , Katz MA , Hernán MA , Lipsitch M , Reis B , Balicer RD . 2021. BNT162b2 mRNA Covid-19 vaccine in a nationwide mass vaccination setting. N Engl J Med 384:1412–1423. doi:10.1056/NEJMoa2101765 33626250 PMC7944975

[3] Thompson MG , Burgess JL , Naleway AL , Tyner HL , Yoon SK , Meece J , Olsho LEW , Caban-Martinez AJ , Fowlkes A , Lutrick K , Kuntz JL , Dunnigan K , Odean MJ , Hegmann KT , Stefanski E , Edwards LJ , Schaefer-Solle N , Grant L , Ellingson K , Groom HC , Zunie T , Thiese MS , Ivacic L , Wesley MG , Lamberte JM , Sun X , Smith ME , Phillips AL , Groover KD , Yoo YM , Gerald J , Brown RT , Herring MK , Joseph G , Beitel S , Morrill TC , Mak J , Rivers P , Harris KM , Hunt DR , Arvay ML , Kutty P , Fry AM , Gaglani M . 2021. Interim estimates of vaccine effectiveness of BNT162b2 and mRNA-1273 COVID-19 vaccines in preventing SARS-CoV-2 infection among health care personnel, first responders, and other essential and frontline workers - eight U.S locations, December 2020-March 2021. MMWR Morb Mortal Wkly Rep 70:495–500. doi:10.15585/mmwr.mm7013e3 33793460 PMC8022879

[4] Shuai H , Chan J-W , Hu B , Chai Y , Yuen T-T , Yin F , Huang X , Yoon C , Hu J-C , Liu H , Shi J , Liu Y , Zhu T , Zhang J , Hou Y , Wang Y , Lu L , Cai J-P , Zhang AJ , Zhou J , Yuan S , Brindley MA , Zhang B-Z , Huang J-D , To K-W , Yuen K-Y , Chu H . 2022. Attenuated replication and pathogenicity of SARS-CoV-2 B.1.1.529 Omicron. Nature 603:693–699. doi:10.1038/s41586-022-04442-5 35062016

[5] Tegally H , Moir M , Everatt J , Giovanetti M , Scheepers C , Wilkinson E , Subramoney K , Moyo S , Amoako DG , Baxter C , Althaus CL , Anyaneji UJ , Kekana D , Viana R , Giandhari J , Lessells RJ , Maponga T , Maruapula D , Choga W , Matshaba M , Mayaphi S , Mbhele N , Mbulawa MB , Msomi N , Naidoo Y , Pillay S , Sanko TJ , San JE , Scott L , Singh L , Magini NA , Smith-Lawrence P , Stevens W , Dor G , Tshiabuila D , Wolter N , Preiser W , Treurnicht FK , Venter M , Davids M , Chiloane G , Mendes A , McIntyre C , O’Toole A , Ruis C , Peacock TP , Roemer C , Williamson C , Pybus OG , Bhiman J , Glass A , Martin DP , Rambaut A , Gaseitsiwe S , Gottberg A von , de Oliveira T , NGS-SA consortium . 2022. Continued emergence and evolution of Omicron in South Africa: new BA.4 and BA.5 lineages. medRxiv. doi:10.1101/2022.05.01.22274406

[6] comercio E . 2022. Confirmada la existencia de una variante asturiana del coronavirus, que pasa a llamarse BF.1. El comercio.

[7] News TM 2022. Another divergent BA.5 subvariant designated as BF.1 has emerged! p In Thailand Medical News.

[8] Ferasin L , Fritz M , Ferasin H , Becquart P , Legros V , Leroy EM . 2021. Myocarditis in naturally infected pets with the British variant of COVID-19. bioRxiv. doi:10.1101/2021.03.18.435945

[9] Barroso-Arévalo S , Sánchez-Morales L , Pérez-Sancho M , Domínguez L , Sánchez-Vizcaíno JM . 2022. First detection of SARS-CoV-2 B.1.617.2 (Delta) variant of concern in a symptomatic cat in Spain. Front Vet Sci 9:841430. doi:10.3389/fvets.2022.841430 35433922 PMC9011004

[10] Fernández-Bastit L , Rodon J , Pradenas E , Marfil S , Trinité B , Parera M , Roca N , Pou A , Cantero G , Lorca-Oró C , Carrillo J , Izquierdo-Useros N , Clotet B , Noguera-Julián M , Blanco J , Vergara-Alert J , Segalés J . 2021. First detection of SARS-CoV-2 Delta (B.1.617.2) variant of concern in a dog with clinical signs in Spain. Viruses 13:2526. doi:10.3390/v13122526 34960795 PMC8704391

[11] Barroso-Arévalo S , Rivera B , Domínguez L , Sánchez-Vizcaíno JM . 2021. First detection of SARS-CoV-2 B.1.1.7 variant of concern in an asymptomatic dog in Spain. Viruses 13:1379. doi:10.3390/v13071379 34372585 PMC8310032

[12] Sánchez-Morales L , Sánchez-Vizcaíno JM , Pérez-Sancho M , Domínguez L , Barroso-Arévalo S . 2022. The Omicron (B.1.1.529) SARS-CoV-2 variant of concern also affects companion animals. Front Vet Sci 9:940710. doi:10.3389/fvets.2022.940710 36032286 PMC9411866

[13] Barroso-Arévalo S , Barneto A , Ramos ÁM , Rivera B , Sánchez R , Sánchez-Morales L , Pérez-Sancho M , Buendía A , Ferreras E , Ortiz-Menéndez JC , Moreno I , Serres C , Vela C , Risalde MÁ , Domínguez L , Sánchez-Vizcaíno JM . 2022. Large-scale study on virological and serological prevalence of SARS-CoV-2 in cats and dogs in Spain. Transbound Emerg Dis 69:e759–e774. doi:10.1111/tbed.14366 34724350 PMC8661836

[14] Perera RAPM , Ko R , Tsang OTY , Hui DSC , Kwan MYM , Brackman CJ , To EMW , Yen H-L , Leung K , Cheng SMS , Chan KH , Chan KCK , Li K-C , Saif L , Barrs VR , Wu JT , Sit THC , Poon LLM , Peiris M . 2021. Evaluation of a SARS-CoV-2 surrogate virus neutralization test for detection of antibody in human, canine, cat, and hamster sera. J Clin Microbiol 59:e02504-20. doi:10.1128/JCM.02504-20 33139421 PMC8111130

[15] Barroso-Arévalo S , Sánchez-Morales L , Barasona JA , Rivera B , Sánchez R , Risalde MA , Agulló-Ros I , Sánchez-Vizcaíno JM . 2022. Evaluation of the clinical evolution and transmission of SARS-CoV-2 infection in cats by simulating natural routes of infection. Vet Res Commun 46:837–852. doi:10.1007/s11259-022-09908-5 35243589 PMC8893356

[16] Fujiki R , Ikeda M , Yoshida A , Akiko M , Yao Y , Nishimura M , Matsushita K , Ichikawa T , Tanaka T , Morisaki H , Morisaki T , Ohara O . 2018. Assessing the accuracy of variant detection in cost-effective gene panel testing by next-generation sequencing. J Mol Diagn 20:572–582. doi:10.1016/j.jmoldx.2018.04.004 29953964

[17] Kumar S , Stecher G , Li M , Knyaz C , Tamura K . 2018. MEGA X: molecular evolutionary genetics analysis across computing platforms. Mol Biol Evol 35:1547–1549. doi:10.1093/molbev/msy096 29722887 PMC5967553

[18] Hall B. 2011. Phylogenetic trees made easy. a how-to manual, 4th edn. ed.

[19] Pejaver V , Urresti J , Lugo-Martinez J , Pagel KA , Lin GN , Nam H-J , Mort M , Cooper DN , Sebat J , Iakoucheva LM , Mooney SD , Radivojac P . 2020. Inferring the molecular and phenotypic impact of amino acid variants with MutPred2. Nat Commun 11:5918. doi:10.1038/s41467-020-19669-x 33219223 PMC7680112

[20] Tamura K . 1992. Estimation of the number of nucleotide substitutions when there are strong transition-transversion and G+C-content biases. Mol Biol Evol 9:678–687. doi:10.1093/oxfordjournals.molbev.a040752 1630306

[21] Patterson EI , Elia G , Grassi A , Giordano A , Desario C , Medardo M , Smith SL , Anderson ER , Prince T , Patterson GT , Lorusso E , Lucente MS , Lanave G , Lauzi S , Bonfanti U , Stranieri A , Martella V , Solari Basano F , Barrs VR , Radford AD , Agrimi U , Hughes GL , Paltrinieri S , Decaro N . 2020. Evidence of exposure to SARS-CoV-2 in cats and dogs from households in Italy. Nat Commun 11:6231. doi:10.1038/s41467-020-20097-0 33277505 PMC7718263

[22] Hamer SA , Pauvolid-Corrêa A , Zecca IB , Davila E , Auckland LD , Roundy CM , Tang W , Torchetti M , Killian ML , Jenkins-Moore M , Mozingo K , Akpalu Y , Ghai RR , Spengler JR , Behravesh CB , Fischer RSB , Hamer GL . 2020. Natural SARS-CoV-2 infections, including virus isolation, among serially tested cats and dogs in households with confirmed human COVID-19 cases in Texas, USA. bioRxiv:2020.12.08.416339. doi:10.1101/2020.12.08.416339 PMC8159091 34069453

[23] Gortázar C , Barroso-Arévalo S , Ferreras-Colino E , Isla J , de la Fuente G , Rivera B , Domínguez L , de la Fuente J , Sánchez-Vizcaíno JM . 2021. Natural SARS-CoV-2 infection in kept ferrets, Spain. Emerg Infect Dis 27:1994–1996. doi:10.3201/eid2707.210096 34152974 PMC8237878

[24] Tegally H , Moir M , Everatt J , Giovanetti M , Scheepers C , Wilkinson E , Subramoney K , Makatini Z , Moyo S , Amoako DG , Baxter C , Althaus CL , Anyaneji UJ , Kekana D , Viana R , Giandhari J , Lessells RJ , Maponga T , Maruapula D , Choga W , Matshaba M , Mbulawa MB , Msomi N , NGS-SA consortium, Naidoo Y , Pillay S , Sanko TJ , San JE , Scott L , Singh L , Magini NA , Smith-Lawrence P , Stevens W , Dor G , Tshiabuila D , Wolter N , Preiser W , Treurnicht FK , Venter M , Chiloane G , McIntyre C , O’Toole A , Ruis C , Peacock TP , Roemer C , Kosakovsky Pond SL , Williamson C , Pybus OG , Bhiman JN , Glass A , Martin DP , Jackson B , Rambaut A , Laguda-Akingba O , Gaseitsiwe S , von Gottberg A , de Oliveira T . 2022. Emergence of SARS-CoV-2 Omicron lineages BA.4 and BA.5 in South Africa. Nat Med 28:1785–1790. doi:10.1038/s41591-022-01911-2 35760080 PMC9499863

[25] Centro de Coordinación de Alertas y Emergencias Sanitarias MdSdE . 2022. Actualización de la situación epidemiológica delas variantes de SARS-CoV-2 en España

[26] Tamura T , Yamasoba D , Oda Y , Ito J , Kamasaki T , Nao N , Hashimoto R , Fujioka Y , Suzuki R , Wang L , Ito H , Kimura I , Yokota I , Kishimoto M , Tsuda M , Sawa H , Yoshimatsu K , Ohba Y , Yamamoto Y , Nagamoto T , Kanamune J , Matsuno K , Takayama K , Tanaka S , Sato K , Fukuhara T , The Genotype to Phenotype Japan (G2P-Japan) Consortium . 2022. Comparative pathogenicity of SARS-CoV-2 Omicron subvariants including BA.1, BA.2, and BA.5. bioRxiv. doi:10.1101/2022.08.05.502758 PMC10366110 37488344

[27] Abdelnabi R , Foo CS , Zhang X , Lemmens V , Maes P , Slechten B , Raymenants J , André E , Weynand B , Dallmeier K , Neyts J . 2022. The Omicron (B.1.1.529) SARS-CoV-2 variant of concern does not readily infect Syrian hamsters. Antiviral Res. 198:105253. doi:10.1016/j.antiviral.2022.105253 35066015 PMC8776349

[28] WHO . 2020. SARS-CoV-2 mink-associated variant strain – Denmark. Available from: https://www.who.int/csr/don/06-november-2020-mink-associated-sars-cov2-denmark/en

[29] Tan CCS , Lam SD , Richard D , Owen CJ , Berchtold D , Orengo C , Nair MS , Kuchipudi SV , Kapur V , van Dorp L , Balloux F . 2022. Transmission of SARS-CoV-2 from humans to animals and potential host adaptation. Nat Commun 13:2988. doi:10.1038/s41467-022-30698-6 35624123 PMC9142586

[30] Candido DS , Claro IM , de Jesus JG , Souza WM , Moreira FRR , Dellicour S , Mellan TA , du Plessis L , Pereira RHM , Sales FCS , Manuli ER , Thézé J , Almeida L , Menezes MT , Voloch CM , Fumagalli MJ , Coletti TM , da Silva CAM , Ramundo MS , Amorim MR , Hoeltgebaum HH , Mishra S , Gill MS , Carvalho LM , Buss LF , Prete CA , Ashworth J , Nakaya HI , Peixoto PS , Brady OJ , Nicholls SM , Tanuri A , Rossi ÁD , Braga CKV , Gerber AL , de C Guimarães AP , Gaburo N , Alencar CS , Ferreira ACS , Lima CX , Levi JE , Granato C , Ferreira GM , Francisco RS , Granja F , Garcia MT , Moretti ML , Perroud MW , Castiñeiras TMPP , Lazari CS , Hill SC , de Souza Santos AA , Simeoni CL , Forato J , Sposito AC , Schreiber AZ , Santos MNN , de Sá CZ , Souza RP , Resende-Moreira LC , Teixeira MM , Hubner J , Leme PAF , Moreira RG , Nogueira ML , Ferguson NM , Costa SF , Proenca-Modena JL , Vasconcelos ATR , Bhatt S , Lemey P , Wu C-H , Rambaut A , Loman NJ , Aguiar RS , Pybus OG , Sabino EC , Faria NR , Brazil-UK Centre for Arbovirus Discovery, Diagnosis, Genomics and Epidemiology (CADDE) Genomic Network . 2020. Evolution and epidemic spread of SARS-CoV-2 in Brazil. Science 369:1255–1260. doi:10.1126/science.abd2161 32703910 PMC7402630

[31] Braun KM , Moreno GK , Halfmann PJ , Hodcroft EB , Baker DA , Boehm EC , Weiler AM , Haj AK , Hatta M , Chiba S , Maemura T , Kawaoka Y , Koelle K , O’Connor DH , Friedrich TC . 2021. Transmission of SARS-Cov-2 in domestic cats imposes a narrow bottleneck. bioRxiv:2020.11.16.384917. doi:10.1101/2020.11.16.384917 PMC7946358 33635912

[32] Bashor L , Gagne RB , Bosco-Lauth AM , Bowen RA , Stenglein M , VandeWoude S . 2021. SARS-CoV-2 evolution in animals suggests mechanisms for rapid variant selection. Proc Natl Acad Sci U S A 118:e2105253118. doi:10.1073/pnas.2105253118 34716263 PMC8612357

[33] Klaus J , Meli ML , Willi B , Nadeau S , Beisel C , Stadler T , Egberink H , Zhao S , Lutz H , Riond B , Rösinger N , Stalder H , Renzullo S , Hofmann-Lehmann R . 2021. Detection and genome sequencing of SARS-CoV-2 in a domestic cat with respiratory signs in Switzerland. Viruses 13:496. doi:10.3390/v13030496 33802899 PMC8002591

[34] Mishra SK , Tripathi T . 2021. One year update on the COVID-19 pandemic: where are we now? Acta Trop 214:105778. doi:10.1016/j.actatropica.2020.105778 33253656 PMC7695590

[35] Gao Y , Yan L , Huang Y , Liu F , Zhao Y , Cao L , Wang T , Sun Q , Ming Z , Zhang L , Ge J , Zheng L , Zhang Y , Wang H , Zhu Y , Zhu C , Hu T , Hua T , Zhang B , Yang X , Li J , Yang H , Liu Z , Xu W , Guddat LW , Wang Q , Lou Z , Rao Z . 2020. Structure of the RNA-dependent RNA polymerase from COVID-19 virus. Science 368:779–782. doi:10.1126/science.abb7498 32277040 PMC7164392

[36] Reshamwala SMS , Likhite V , Degani MS , Deb SS , Noronha SB . 2021. Mutations in SARS-CoV-2 nsp7 and nsp8 proteins and their predicted impact on replication/transcription complex structure. J Med Virol 93:4616–4619. doi:10.1002/jmv.26791 33433004 PMC8012999

[37] Chen J , Malone B , Llewellyn E , Grasso M , Shelton PMM , Olinares PDB , Maruthi K , Eng ET , Vatandaslar H , Chait BT , Kapoor TM , Darst SA , Campbell EA . 2020. Structural basis for helicase-polymerase coupling in the SARS-CoV-2 replication-transcription complex. Cell 182:1560–1573. doi:10.1016/j.cell.2020.07.033 32783916 PMC7386476

[38] Schmidt SM . 2020. The role of iron in viral infections. Front Biosci (Landmark Ed) 25:893–911. doi:10.2741/4839 31585922

[39] Fillebeen C , Pantopoulos K . 2010. Iron inhibits replication of infectious hepatitis C virus in permissive Huh7.5.1 cells. J Hepatol 53:995–999. doi:10.1016/j.jhep.2010.04.044 20813419

[40] Patel PH , Loeb LA . 2000. DNA polymerase active site is highly mutable: evolutionary consequences. Proc Natl Acad Sci U S A 97:5095–5100. doi:10.1073/pnas.97.10.5095 10805772 PMC25787

